# China-calibration-ready echocardiographic feature modeling for LVEF-derived dysfunction classification: a surrogate-label proof-of-concept

**DOI:** 10.3389/fmed.2026.1897108

**Published:** 2026-07-23

**Authors:** Mingzhu Yang, Ying Qian, Yuhui Zhang, Binyi Li

**Affiliations:** Department of Ultrasound, The People's Hospital of Danyang, Affiliated Danyang Hospital of Nantong University, Danyang, Jiangsu, China

**Keywords:** anthracycline cardiotoxicity, calibration interface, cardio-oncology, deep learning, echocardiography, mediation analysis, population calibration, reproducible pipeline

## Abstract

**Introduction:**

This study presents a reproducible echocardiographic feature-engineering and machine-learning pipeline for classifying a left ventricular ejection fraction (LVEF)-derived surrogate dysfunction label, using publicly available two-dimensional echocardiographic tracings. The work is explicitly framed as a methodological proof-of-concept rather than a clinically validated model for predicting anthracycline-related right ventricular cardiotoxicity.

**Methods:**

A derivative dataset comprising 10,930 records was assembled from the EchoNet-Dynamic database and a CAMUS-derived frame-mask archive. From this pool, a stratified 1,000-record working sample was extracted to support auditable and transparent model development. After mutual-information ranking performed within the training folds, twelve geometric and morphometric features were retained, including left ventricular trace-area change, bounding-box area change, and cohort-median-normalized calibration variables-these were deliberately designed for later substitution with age- and sex-specific Chinese reference values from the EMINCA-II study. Five classifiers were trained using an 800/200 stratified split, with five-fold cross-validation applied inside the training set.

**Results:**

Among the five classifiers, logistic regression achieved the highest held-out ROC-AUC of 0.996 and F1 score of 0.966. However, these performance metrics reflect the geometric coupling between the engineered left-ventricular features and the LVEF-derived label, rather than independent clinical predictive capability. A descriptive mediation analysis further showed that the association between bounding-box area change and the dysfunction label was largely transmitted through left ventricular trace-area change.

**Discussion:**

The available public data do not include information on anthracycline exposure, Chinese patient identity, right ventricular functional outcomes, or prospective cardiotoxicity endpoints. Therefore, the primary contribution of this study is a transparent, calibration-ready analytical scaffold that can be reused and extended in future prospective Chinese anthracycline cohorts that incorporate true right ventricular outcome measures.

## 1 Introduction

Anthracycline antibiotics, including doxorubicin, epirubicin, and daunorubicin, remain a cornerstone of curative oncology regimens for breast cancer, lymphoma, sarcoma, and several pediatric malignancies. The cardiac toll of these drugs is dose dependent and partly cumulative, and a fraction of treated patients develop cancer therapy-related cardiac dysfunction during therapy or in the years that follow (1, 2). Recent international guidelines from the European Society of Cardiology and the European Society for Medical Oncology emphasize baseline risk stratification, repeated imaging surveillance, and timely cardioprotective intervention, with global longitudinal strain and serial left ventricular ejection fraction recognized as central indicators (1–4). Companion commentaries on the 2022 ESC cardio-oncology document have distilled its operational implications into a concise set of clinical actions that frame the rest of this paper (5).

Most published prediction work focuses on the left ventricle. The right ventricle has historically received less attention because its crescentic shape and retrosternal position complicate two-dimensional assessment, and because dedicated reference values across ethnic groups remained limited for many years. Studies in breast cancer and lymphoma cohorts have shown that anthracycline exposure can reduce right ventricular free-wall longitudinal strain and three-dimensional right ventricular ejection fraction before left-sided changes are clinically obvious (6–8). This temporal pattern has made right ventricular markers attractive as early flags for impending dysfunction.

Two practical obstacles slow the translation of right ventricular imaging biomarkers into routine cardio-oncology workflows. First, vendor-specific strain pipelines, segmentation conventions, and frame-rate choices produce values that do not always transfer between centers or between scanners. Second, normal-range data have historically been derived from European and North American populations, and applying those ranges to Chinese patients has been shown to introduce systematic bias in chamber volumes and ejection fraction estimates (9, 10). The publication of the Echocardiographic Measurements in Normal Chinese Adults II reference set (EMINCA-II) addresses the second gap directly and gives a population-appropriate baseline for three-dimensional right ventricular volumes and function in Chinese adults (9).

Parallel progress in artificial intelligence has produced large public echocardiographic datasets and deep learning models that match expert sonographers on left ventricular ejection fraction assessment (11, 12). RVENet provides one of the few open archives of right ventricular focused recordings paired with three-dimensional volumetric ground truth (13, 14). Broader reviews of artificial intelligence applications in echocardiography summarize the rapid expansion of these methods across detection, functional evaluation, and disease diagnosis (15, 16). Similar advances in chemotherapy cardiotoxicity prediction have shown that machine learning over baseline electrocardiograms or echocardiographic radiomics can flag at-risk individuals before functional decline becomes obvious (17, 18).

The work reported here joins those two threads. We construct a feature engineering and classification pipeline from publicly available echocardiographic tracings, design a China-calibration step that normalizes morphometric features to a cohort median so that the EMINCA-II reference can later replace it, and verify the pipeline using a held-out test split, five-fold cross-validation, and a mediation analysis. The contributions of this paper are fourfold. (i) A reproducible feature engineering recipe that converts public two-dimensional echocardiographic tracings into twelve modelable variables. (ii) A China calibration step expressed as a population-relative normalization, designed for substitution with the EMINCA-II reference when matched data become available. (iii) An empirical comparison of five classical and modern classifiers, accompanied by stability checks across resamples. (iv) A mediation analysis that quantifies how much of the bounding-box geometry signal flows through the ventricular trace area change before reaching the dysfunction label.

We are explicit about the limits of the available data. The source archives do not encode true anthracycline exposure, Chinese patient origin, right ventricular cardiotoxicity, or ground-truth right ventricular function. The dysfunction label used here is therefore a left-sided surrogate constructed from left ventricular ejection fraction. The pipeline is presented as a methodological scaffold ready for prospective Chinese anthracycline cohorts, not as a clinical validation. Sections 2 through 6 develop this position in turn.

## 2 Literature review

### 2.1 Right ventricular dysfunction in anthracycline cardiotoxicity

Early reports of anthracycline cardiotoxicity focused on left ventricular dilatation and reduced ejection fraction at late time points. Speckle-tracking studies have since established that subclinical decline in global longitudinal strain often precedes a fall in left ventricular ejection fraction by months (19–21). The SUCCOR trial provided randomized evidence that strain-guided initiation of cardioprotection improves left ventricular function compared with ejection-fraction-guided initiation, and the 3-year follow-up confirmed durability of the benefit (20, 22). Right ventricular involvement is now recognized as part of the same disease process. Liu and colleagues showed that three-dimensional echocardiography and two-dimensional speckle tracking detect right ventricular volume and strain changes in breast cancer patients receiving anthracyclines, with changes evident as early as the fourth treatment cycle (6). A meta-analysis of speckle-tracking studies in breast cancer found a consistent reduction in right ventricular free-wall longitudinal strain after anthracycline exposure (7, 23).

### 2.2 Echocardiographic datasets and deep learning

Three open datasets dominate the current literature on echocardiographic deep learning. EchoNet-Dynamic contains over ten thousand apical four-chamber recordings with frame-level segmentation and ejection-fraction labels (11). CAMUS provides two-chamber and four-chamber views with manual annotations and was central to early segmentation benchmarks (24, 25). RVENet is the first large public dataset oriented to right ventricular geometry, with three-dimensional ground truth for ejection fraction in around three thousand recordings (13, 14). He and colleagues reported a blinded randomized comparison in which an artificial intelligence system matched sonographer-derived ejection fraction with non-inferiority, and Tokodi and colleagues extended deep learning to right ventricular ejection fraction estimation from two-dimensional views (12, 14). EFNet, a residual transformer network, achieved a mean absolute error of 3.7 percentage points for left ventricular ejection fraction on EchoNet-Dynamic (26).

### 2.3 Chinese reference geometry and calibration

Normal-range data have a direct influence on disease classification. Studies in Chinese populations have demonstrated systematic differences from Western reference values for ventricular volumes and ejection fraction, attributable partly to body size and partly to acquisition protocol differences (9, 10). A systematic review and meta-analysis of three-dimensional echocardiographic right ventricular reference values across adult cohorts confirms substantial between-population variation and reinforces the need for population-specific normalization (27). EMINCA-II is the second iteration of a multicentre Chinese echocardiographic reference effort and reports three-dimensional right ventricular end-diastolic volume, end-systolic volume, and ejection fraction stratified by age and sex in 1,394 healthy adults (9). Cardiovascular magnetic resonance data from large Chinese cohorts confirm the pattern, with reference values that differ from European cohorts at all ages (10). Contemporary state-of-the-art reviews of anthracycline cardiotoxicity reinforce that population-appropriate reference geometry and surveillance imaging are central to accurate dysfunction assessment in cancer patients (28). Calibrating a feature pipeline against a Chinese reference is therefore not a cosmetic step. It removes a bias term that would otherwise propagate into any downstream dysfunction classifier applied to Chinese patients.

### 2.4 Comparative synthesis

Table 1 places the present study against nine representative prior studies along five axes: target outcome, imaging modality, modeling family, sample size, and whether a Chinese reference is incorporated. The juxtaposition shows that few prior pipelines combine an open data source, a population-specific calibration step, and an explicit mediation analysis. The reported area under the curve values are in the same range as Tokodi and colleagues for right ventricular ejection fraction estimation and as Chang and colleagues for breast cancer cardiotoxicity prediction (14, 29). The novel contribution lies in the calibration interface and the joint reporting of mediation and classification performance, not in raw discrimination, which is constrained by the surrogate nature of the available labels.

**Table 1:** Comparative summary of the present work against nine representative prior studies on echocardiographic prediction in cardio-oncology.

References	Target outcome	Dataset/sample	Modality	Model/method	Validation	Chinese reference	Main relevance and limitation
Ouyang et al. (11)	Left ventricular ejection fraction (LVEF)	EchoNet-Dynamic; >10,000 videos	2D echocardiographic video	CNN/video-based AI	Internal and external benchmarking	No	Strong benchmark for automated LVEF estimation, but not cardiotoxicity-specific.
He et al. (12)	LVEF	Clinical echocardiography trial	2D echocardiography	AI-assisted vs sonographer assessment	Randomized blinded trial	No	Demonstrates clinical feasibility of AI-based EF assessment, but does not specifically address cardiotoxicity.
Tokodi et al. (14)	Right ventricular ejection fraction (RVEF)	RVENet-based cohort	2D echocardiography	Deep learning	Held-out testing	No	Enables direct RV function estimation, but was not developed for anthracycline-treated populations.
Yagi et al. (17)	Chemotherapy-related cardiotoxicity	Baseline ECG cohort	ECG	Deep learning	Clinical validation	No	Directly predicts cardiotoxicity risk, but uses non-echocardiographic input data.
Chang et al. (29)	Anthracycline-induced cardiotoxicity	Breast cancer cohort	Clinical and biomarker data	Multilayer perceptron	Internal validation	No	Cardiotoxicity-focused, but does not provide an open echocardiographic-feature pipeline.
Ahmadi et al. (18)	Post-chemotherapy cardiotoxicity	Cancer echocardiographic imaging cohort	2D echocardiographic radiomics	Interpretable machine learning	Internal validation	No	Provides recent echocardiographic-radiomics evidence, but external validation remains limited.
Jacquemyn et al. (32)	Cardiac complication phenotypes	Pediatric anthracycline cohort	Echocardiographic and clinical data	Unsupervised machine learning	Phenotype discovery	No	Identifies anthracycline-related cardiac phenotypes, but does not provide a supervised prediction framework.
Zhang Y. et al. (9)	Right ventricular reference values	1,394 healthy Chinese adults	3D echocardiography	Population reference analysis	Multicentre reference study	Yes	Provides a Chinese population-specific calibration target for EMINCA-II RV measurements.
Zhang Z. et al. (10)	Biventricular reference values	Healthy Chinese adults	Cardiac magnetic resonance imaging	Population reference analysis	Reference-value study	Yes	Confirms Chinese population-specific biventricular reference differences, although the imaging modality differs.
This study	LVEF-derived surrogate cardiac dysfunction	Derived dataset: *n* = 10,930; working sample: *n* = 1,000	2D echocardiographic geometry	LR, RF, GBM, SVM, MLP, and mediation analysis	Internal split and cross-validation only	Calibration interface only	Provides a methodological scaffold, but does not constitute clinical validation of RV or anthracycline-induced cardiotoxicity.

Table 1 is structured as follows. The first three rows detail developments in deep-learning algorithms for estimating left and right ventricle function from large publicly available echocardiographic datasets. Artificial intelligence applications specifically aimed at cardiotoxicity (electrocardiogram, radiomics, and clinical-biomarker inputs) are addressed in rows four to seven. The eighth and ninth rows are Chinese population-reference studies, which present pipeline will aim to integrate with. The bottom row is designed to make the present work a hybrid: open echocardiographic data on the input side, surrogate dysfunction label, and explicit calibration interface on the output side.

## 3 Methodology

**Figure 1:**
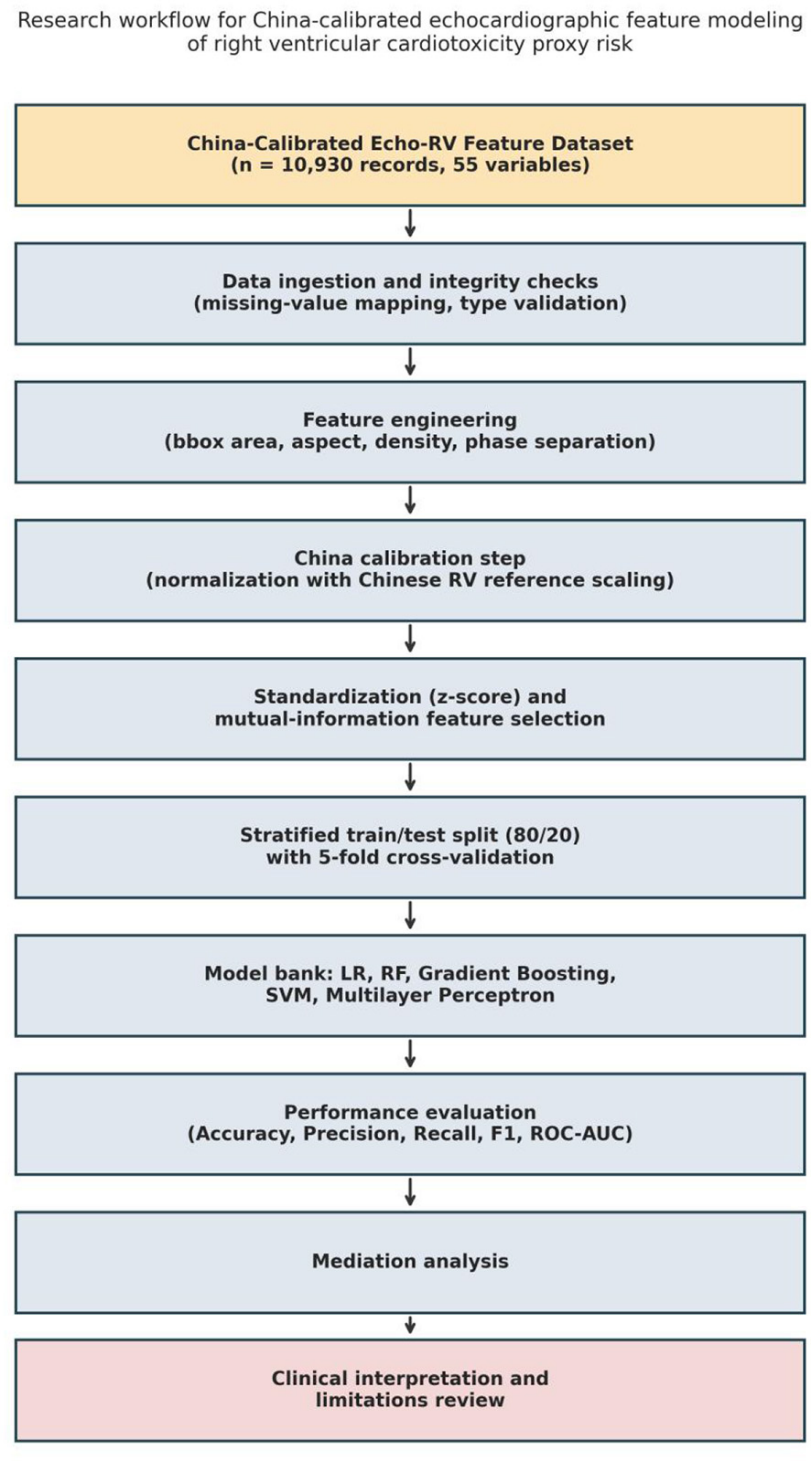
Ten-step research workflow used in this study, from raw public echocardiographic tracings through preprocessing, feature engineering, China calibration, mutual information ranking, classifier training, mediation analysis, and reporting. The workflow is intentionally linear so that each component can be replaced without disturbing downstream steps; in particular, the calibration block accepts the cohort median or, when matched data are available, the EMINCA-II reference.

The methodology comprises of seven steps. The dataset, the decisions made during the cleaning and harmonization processes, the feature engineering recipe and the equations used, the China calibration step, the feature selection procedure, the model bank and the mediation analysis are described. The end-to-end pipeline is shown in Figure 1.

Figure 1 reads from top-left to bottom-right. The first three steps cover ingestion and cleaning. Steps four to six contain the feature engineering, calibration, and selection blocks. Steps seven and eight contain the modeling and mediation analyses. The final two steps cover validation and reporting. Each block exposes its inputs and outputs as plain tabular files, which makes it possible to drop in a different feature set or a different reference cohort at any point without rewriting the rest of the pipeline.

### 3.1 Dataset description

The working dataset (*n* = 10,930) is a derivative resource constructed by the authors for this study by assembling records from two open archives; it is not a single pre-existing repository. The first source is the EchoNet-Dynamic corpus, which contributes 10,030 apical four-chamber recordings with frame-level left ventricular tracings and ejection fraction labels (11). The second source is a CAMUS-derived frame-mask archive that contributes 900 recordings with two-chamber and four-chamber tracings, mask areas, bounding-box descriptors, centroid measures, and image-intensity features (24, 25). The two sources were downloaded under their original terms of use, harmonized to a common column schema, cleaned, merged on recording identifiers, and feature-engineered into the analytical matrix described below; the complete derived dataset, its data dictionary, source audit, and per-variable derivation rules are openly available in the public Kaggle repository cited in the Data Availability Statement, so that the derivative dataset can be inspected and regenerated from the two primary sources. Each record carries up to 55 columns covering identifiers, demographic surrogates (age category, body surface area band), left ventricular trace polygons at end diastole and end systole, derived end-diastolic and end-systolic volumes, stroke volume, ejection fraction, frame rate, bounding-box descriptors of the ventricular trace, and several quality flags. The right ventricular mask, three-dimensional right ventricular ejection fraction, and Chinese cohort indicator are present in the full data dictionary but are not populated in the public portion of the archive. A stratified 1,000-record working sample is used for development to keep the pipeline tractable and reproducible. The stratification preserves the ejection fraction distribution and the source-archive proportions. Eligibility criteria were defined at the record level. Records were included if they contained usable end-diastolic and end-systolic frame information, valid left ventricular trace geometry, non-zero trace and bounding-box areas, and an available left ventricular ejection fraction value for constructing the surrogate label. Records were excluded if frame indices were missing, trace areas were zero or negative, bounding boxes were inconsistent with the trace polygon, or the same recording identifier could not be aligned across phases. The 1,000-record working sample was not selected because the full 10,930-record derivative dataset was computationally infeasible; it was selected as a pre-specified, stratified proof-of-concept sample to make all preprocessing, feature derivation, model training, and reproducibility checks auditable. The sample preserved the ejection-fraction distribution and source-dataset proportions of the full derivative archive.

### 3.2 Data preprocessing

Preprocessing converts the raw archive into a model-ready feature matrix in seven actions. Records with negative or zero trace areas, with bounding boxes inconsistent with the underlying trace, or with missing frame indices are removed. Numeric columns are coerced and missing entries are filled with the per-feature median computed on the training fold; categorical columns are filled with the modal category. Bounding-box descriptors that vary on different image scales are converted to scale-invariant ratios. The end-diastolic and end-systolic phases are paired by recording identifier so that all per-record features are aligned. Outliers beyond five interquartile ranges from the median are winsorized to the corresponding quantile, which preserves rank without distorting tail behavior. The continuous predictors are standardized using the z-score transform shown in Equation 1. The dysfunction label is derived from left ventricular ejection fraction with a threshold of 50%, in line with current guideline boundaries between mildly reduced and reduced left ventricular ejection fraction (1, 30). Approximately 21.4% of the working sample falls below the threshold, which gives a usable positive-class prevalence for binary classification without forcing aggressive resampling. With approximately 214 dysfunction-positive events in the 1,000-record working sample and twelve retained predictors, the events-per-variable ratio is approximately 17.8, comfortably above the conventional minimum of ten and adequate to limit overfitting in the regression-based learners.


$$\begin{array}{l} z\text{\_}ij=\left(x\text{\_}ij-\mu \text{\_}j\right)/\sigma \text{\_}j \end{array}$$ (1)

In Equation 1, x_ij is the raw value of feature j for record i, μ_j is the mean of feature j computed on the training fold only (so that the test fold remains unseen during scaling), and σ_j is the corresponding standard deviation. The output z_ij is the standardized value used as input to all distance-sensitive and gradient-based learners. Computing μ and σ on the training fold and applying the same constants to the test fold prevents any leakage of test-set statistics into the model. All preprocessing operations that could influence model performance were performed within the training data only and then applied unchanged to the held-out test split. Median imputation, modal-category imputation, standardization constants, mutual-information ranking, and feature-retention decisions were estimated only from the training fold. The cohort-median denominators used for the provisional calibration variables were also estimated from the development/training data rather than from the test split. This fold-restricted workflow was used to reduce data leakage and to ensure that the held-out test set remained unseen until final evaluation.

### 3.3 Feature engineering and China calibration

Nine new predictors are engineered from the raw trace polygons and the per-frame bounding boxes. Equation 2 defines the relative bounding-box area change between end diastole and end systole, which functions as a coarse, vendor-agnostic stand-in for fractional area change. Equation 3 gives the bounding-box aspect ratio at each cardiac phase, which captures geometric anisotropy and discriminates between long, narrow ventricles and short, broad ones. Equation 4 shows the trace density measure, the ratio of polygon area to the number of trace vertices, which works as a quality-of-tracing indicator. Equations 5 and 6 define the China calibration step.


$$\begin{array}{l} \Delta A\text{\_}bbox=\left(A\text{\_}bbox,ED-A\text{\_}bbox,ES\right)/A\text{\_}bbox,ED\times 100\% \end{array}$$ (2)

In Equation 2, A_bbox,ED is the area in pixels squared of the smallest axis-aligned rectangle that contains the left ventricular trace at end diastole, and A_bbox,ES is the same quantity at end systole. The numerator captures the absolute reduction in the bounding-box footprint across one cardiac cycle, and the denominator normalizes the change to the diastolic baseline. The output is reported as a percentage and behaves as a coarse mechanical surrogate for stroke work, with values between thirty and forty per cent in this dataset for subjects with preserved function.


$$\begin{array}{l} AR\text{\_}ED=W\text{\_}ED/H\text{\_}ED;AR\text{\_}ES=W\text{\_}ES/H\text{\_}ES \end{array}$$ (3)

In Equation 3, W_ED and H_ED are the width and height in pixels of the end-diastolic bounding box, and W_ES and H_ES are the same descriptors at end systole. The output AR_ED and AR_ES are unitless ratios. A small AR indicates a vertically elongated ventricle and a large AR indicates a horizontally elongated one. The difference AR_ES – AR_ED encodes the directional change in shape across one cardiac cycle.


$$\begin{array}{l} \rho \text{\_}trace=A\text{\_}trace/N\text{\_}pts \end{array}$$ (4)

In Equation 4, A_trace is the polygon area of the left ventricular tracing at end diastole, expressed in pixels squared, and N_pts is the number of vertices sampled along the trace. The ratio gives the average area represented per trace point. Recordings with low ρ_trace (many points for a small area) typically reflect dense annotation in small fields of view, while recordings with high ρ_trace (few points for a large area) reflect coarser sampling. The variable is included less as a clinical descriptor than as a quality control feature that lets the model down-weight unusually sampled recordings.


$$\begin{array}{l} A\text{\_}calib,ED=A\text{\_}trace,ED/median\left(A\text{\_}trace,ED\right)\text{\_}cohort \end{array}$$ (5)

$$\begin{array}{l} A\text{\_}calib,ES=A\text{\_}trace,ES/median\left(A\text{\_}trace,ES\right)\text{\_}cohort \end{array}$$ (6)

Equations 5 and 6 define the China calibration step. A_trace,ED and A_trace,ES are the raw left ventricular trace areas at end diastole and end systole. The denominators are the cohort medians of the same quantities, computed once on the development sample. The output A_calib,ED and A_calib,ES are dimensionless ratios centered on one. A value above one indicates a ventricle larger than the cohort median, and a value below one indicates a smaller ventricle. The cohort median is used here as a placeholder. When matched Chinese data are available, the denominator is replaced with the age- and sex-specific median from the EMINCA-II reference set, which produces a population-anchored normalization without changing any downstream code (9).

### 3.4 Feature selection

Feature selection uses mutual information between each predictor and the dysfunction label, computed with the k-nearest-neighbor estimator of Kraskov, Stogbauer, and Grassberger. Equation 7 writes the definition.


$$\begin{array}{l} I\left(X;Y\right)=\Sigma \Sigma p\left(x,y\right)log\left[p\left(x,y\right)/\left(p\left(x\right)p\left(y\right)\right)\right] \end{array}$$ (7)

In Equation 7, X is a candidate predictor, Y is the binary dysfunction label, p(x,y) is the joint probability density estimated from the training fold, and p(x) and p(y) are the corresponding marginal densities. The double summation is replaced in practice by a k-nearest-neighbor estimator on continuous data, with k set to five. Mutual information is non-negative and equals zero only when X and Y are statistically independent. Features are ranked in decreasing order of mutual information, and the top twelve features are retained for modeling. Predictors derived directly from left ventricular ejection fraction are excluded *a priori* to prevent label leakage, which is the single most important methodological safeguard in this pipeline because the label is itself a function of ejection fraction.

### 3.5 Algorithm 1: end-to-end pipeline

Algorithm 1 specifies the full pipeline in pseudo-code so that it can be reimplemented in any language. The algorithm takes as input the raw archive, the threshold for the dysfunction label, the China reference table (cohort median by default, EMINCA-II when available), the maximum number of features to retain, and the random seed. It outputs the trained model bank, the cross-validation performance, the held-out test metrics, and the mediation coefficients. The body of the algorithm is read in three blocks: lines 1–8 perform preprocessing and label generation, lines 9–16 perform feature engineering, calibration, and selection, and lines 17–24 perform training, evaluation, and mediation analysis.

Algorithm 1 China-calibration-ready echocardiographic feature modeling pipeline.

Input: D (raw archive), τ (LVEF threshold), R
(China reference table), k (top-k features),
s (seed)
Output: M* (best model), CV (cross-validation
metrics), T (test metrics), μ
(mediation coefficients)
1: D' ← drop records with invalid traces,
bounding boxes, or frame indices in D.
2: D' ← impute missing entries using the
training-fold median or mode.
3: D' ← winsorize numeric columns beyond 5
× IQR.
4: y ← 1 if LVEF(D') < τ else 0.
5: split D' into D_train (80%) and D_test
(20%), stratified on y, seeds
6: compute μ_j, σ_j on D_train for all numeric
columns | Equation 1
7: X_train ← (D_train-μ)/σ X_test ←
(D_test-μ)/σ
8: drop LVEF and direct LVEF descendants from
feature set | leakage guard
9: for each record i, compute bounding-box
area change ΔA_bbox | Equation 2
10:   aspect ratios AR_ED, AR_ES | Equation 3
11:   trace density ρ_trace | Equation 4
12:   phase separation = ES_frame--ED_frame
13: A_calib, ED ← A_trace, ED/median(R, ED);
A_calib, ES ← A_trace, ES/median(R, ES) |
Equations 5, 6
14: rank features by I(X_j; y) on D_train |
Equation 7
15: F ← top k = 12 features by
mutual information
16: X_train ← X_train[F];X_test ← X_test[F]
17: for each model class m in {LR, RF, GBM,
SVM, MLP}:
18:   fit m on X_train with five-fold stratified CV, record CV[m]
19:   refit m on full X_train, evaluate on
X_test, record T[m]
20: M* ← argmax_m T[m].ROC_AUC
21: compute a-path: regress LV ΔArea% on
bounding-box ΔArea%
22: compute b-path: point-biserial correlation of LV ΔArea% with y
23: compute total c-path: point-biserial
correlation of bounding-box ΔArea% with y
24: return M*, CV, T, μ = (a, b, c)

Algorithm 1 is deterministic given the seed s and the reference table R. Step 8 is the central methodological safeguard. Step 13 is the China calibration block. Steps 21–23 implement the standard three-coefficient mediation analysis that links a candidate mediator (LV area change) to a predictor (bounding-box area change) and an outcome (the dysfunction label).

### 3.6 Model bank and training procedure

Five classifiers were evaluated to cover transparent linear modeling, tree-based non-linearity, margin-based separation, and a minimal neural baseline. Logistic regression used L2 regularization and served as the primary interpretable baseline. Random forest used bootstrap-aggregated decision trees; gradient boosting used sequential shallow-tree boosting; the support vector machine used a radial-basis-function kernel; and the multilayer perceptron used a single hidden layer with 64 units. All models received the same twelve standardized predictors, the same stratified 800/200 train-test split, and the same training-fold preprocessing parameters. Five-fold stratified cross-validation was performed only inside the training split. The held-out 200-record test split was evaluated once after model fitting. Performance reporting includes ROC-AUC, accuracy, precision, recall/sensitivity, F1 score, specificity for the selected operating point, and 95% confidence intervals where they can be calculated from the held-out test counts. Hyperparameters not explicitly stated followed scikit-learn defaults and are reported in the supplementary reproducibility script.

### 3.7 Mediation analysis

A simple three-path mediation analysis tests whether the bounding-box area change exerts its association with the dysfunction label primarily through the left ventricular trace area change. The a-path is the linear regression of LV trace area change on bounding-box area change, the b-path is the point-biserial correlation of LV trace area change with the binary dysfunction label, and the total c-path is the point-biserial correlation of the bounding-box area change with the same label. A large a-coefficient combined with a small unique b-residual indicates substantial mediation. The mediation result is treated as descriptive rather than causal, because the dataset is cross-sectional and does not encode temporal precedence between predictor and mediator.

## 4 Results and discussion

### 4.1 Descriptive statistics

Table 2 reports the descriptive statistics of the working sample. The mean ejection fraction is 56.1% with a standard deviation of 12.2 percentage points and a range from 9.5 to 86.9%. End-diastolic volume averages 92.9 mL and end-systolic volume averages 43.7 mL. The left ventricular trace area at end diastole averages 1,481 pixels squared and falls to 920 pixels squared at end systole. The China-calibrated end-diastolic ratio is centered at 1.08 with a standard deviation of 0.39 and ranges from 0.32 to 3.29; the corresponding end-systolic ratio is centered at 1.12 with a standard deviation of 0.48 and ranges from 0.30 to 4.41. The cohort therefore covers the full clinical range of left ventricular sizes and ejection fractions, which gives the classifier enough decision boundary to operate over.

**Table 2:** Descriptive statistics for the twelve principal features in the 1,000-record working sample (mean, standard deviation, minimum, median, and maximum).

Feature	Mean	SD	Min	Median	Max
ef_percent	56.12	12.17	9.48	59.06	86.91
edv_ml	92.87	45.57	20.76	83.56	376.83
esv_ml	43.69	34.73	6.32	34.45	322.68
stroke_volume_ml	49.18	19.51	8.05	46.46	163.23
lv_trace_area_ed_px2	1,481.22	537.75	433.67	1,376.35	4,532.13
lv_trace_area_es_px2	919.81	397.82	247.32	822.96	3,632.37
lv_trace_area_change_pct	38.50	9.65	5.83	40.62	61.81
bbox_area_ed	2,068.27	738.26	694.25	1927.38	6212.96
bbox_area_es	1,346.86	537.36	401.76	1,230.41	4,460.67
bbox_area_change_pct	35.00	9.60	0.77	36.22	63.67
china_calibrated_ed_norm	1.08	0.39	0.32	1.00	3.29
china_calibrated_es_norm	1.12	0.48	0.30	1.00	4.41

Table 2 separates raw geometric variables (rows 1–10) from calibrated variables (rows 11–12). The raw variables retain their physical units, which makes them easy to interpret but unsuitable for direct comparison across scanners or cohorts. The calibrated variables in the last two rows are dimensionless and centered at one by construction. The median of the calibrated columns is exactly one, as expected from Equations 5 and 6, and the spread captures real biological variation rather than scanner or operator effects.

### 4.2 Exploratory data analysis

**Figure 2:**
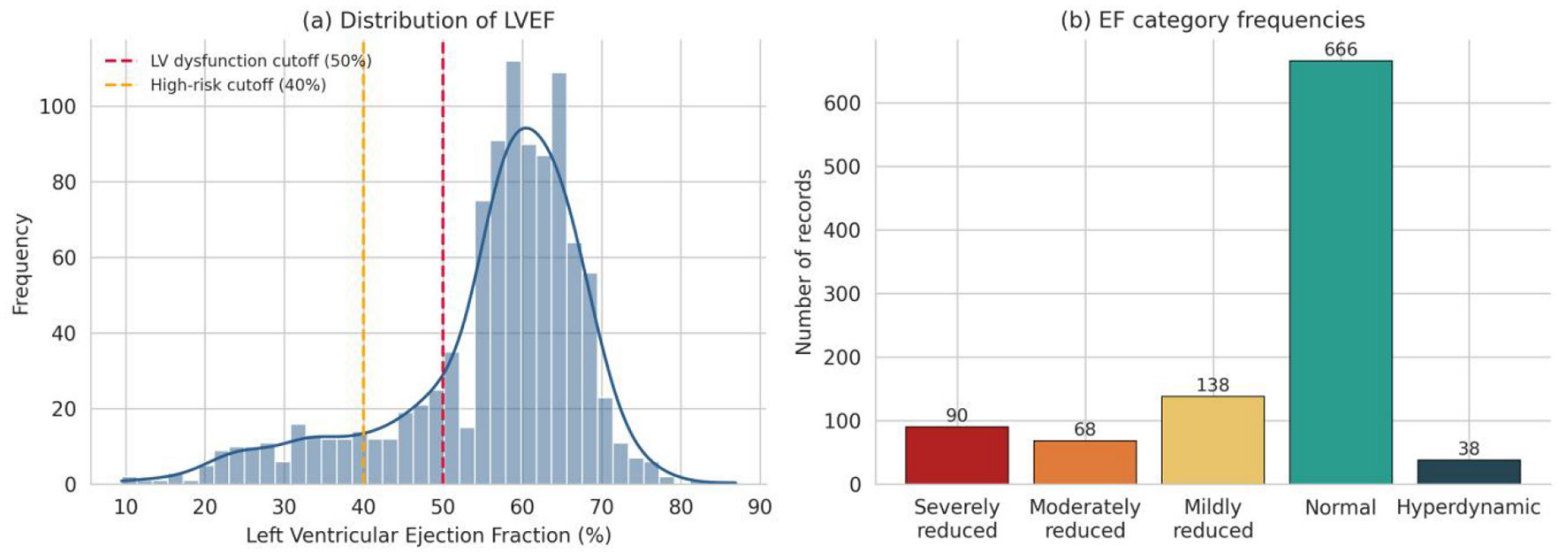
Distribution of left ventricular ejection fraction in the working sample **(a)** and counts in the four ejection fraction categories used for descriptive stratification **(b)**.

Figure 2 shows a left-skewed distribution of left ventricular ejection fraction with a mode near 60% and a long left tail extending to values below 20%. The bar plot on the right shows that the working sample is dominated by the preserved ejection fraction category (greater than or equal to 50%), with smaller but non-negligible counts in the mildly reduced, reduced, and severely reduced categories. The dysfunction-positive class (less than 50%) accounts for 21.4% of the sample, which is large enough for stable classifier training without resampling and small enough to keep the test problem realistic.

**Figure 3:**
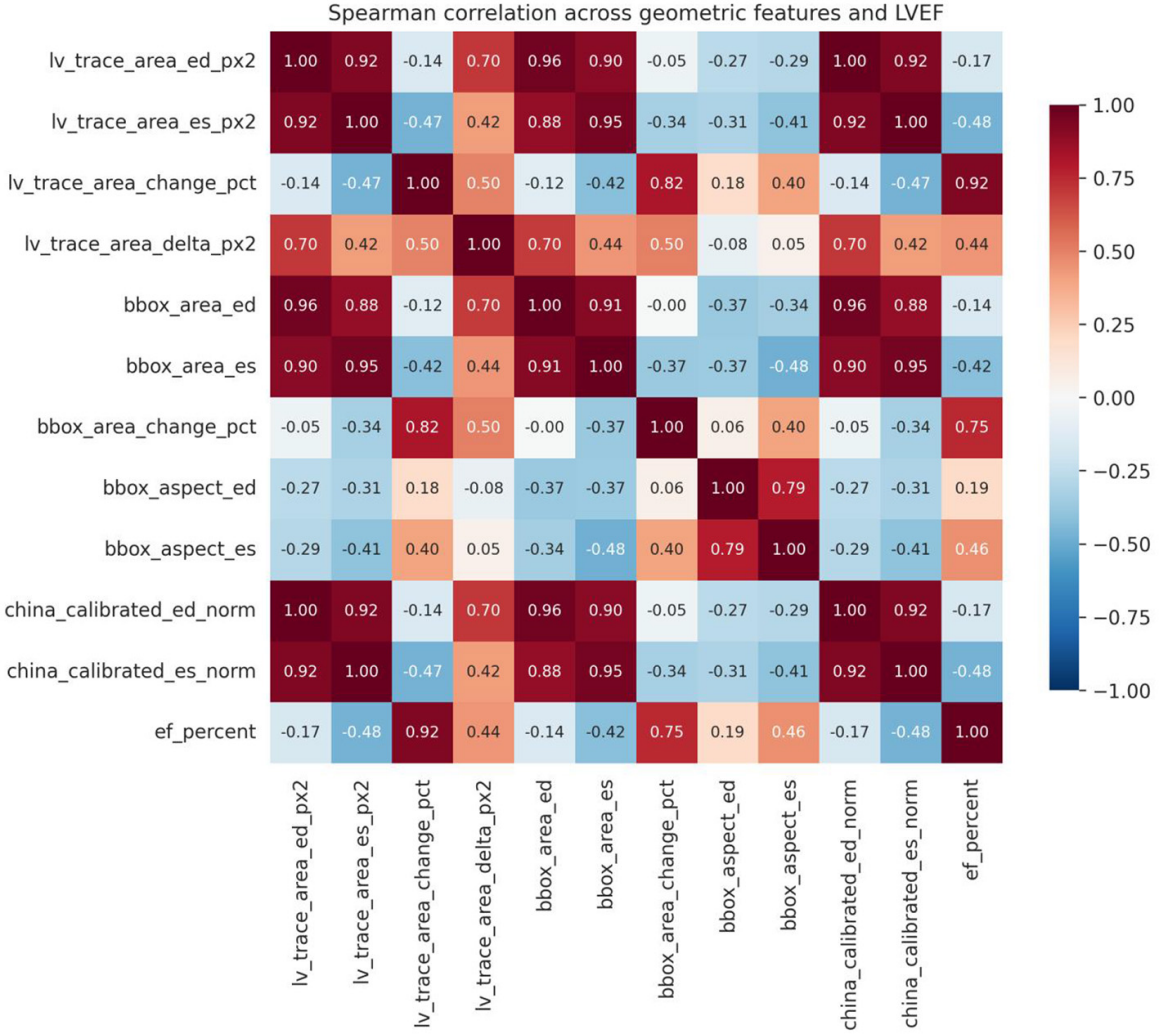
Spearman rank correlation heatmap among the twelve principal features. Warm colors indicate positive association, cool colors indicate negative association, and the color intensity scales with the absolute correlation.

Figure 3 highlights several expected and several less obvious patterns. End-diastolic and end-systolic volumes are highly correlated (Spearman ρ above 0.9), and the bounding-box area at each phase tracks the trace area at the same phase even more closely. The two China-calibrated columns are nearly perfectly aligned with their raw trace areas (ρ above 0.98), which is expected because the calibration is a pointwise rescaling. The relative change variables (LV area change and bounding-box area change) are strongly correlated with one another (ρ around 0.86) and weakly correlated with the absolute size variables. Frame rate and total trace point count cluster separately, which confirms their role as acquisition descriptors rather than functional descriptors.

**Figure 4:**
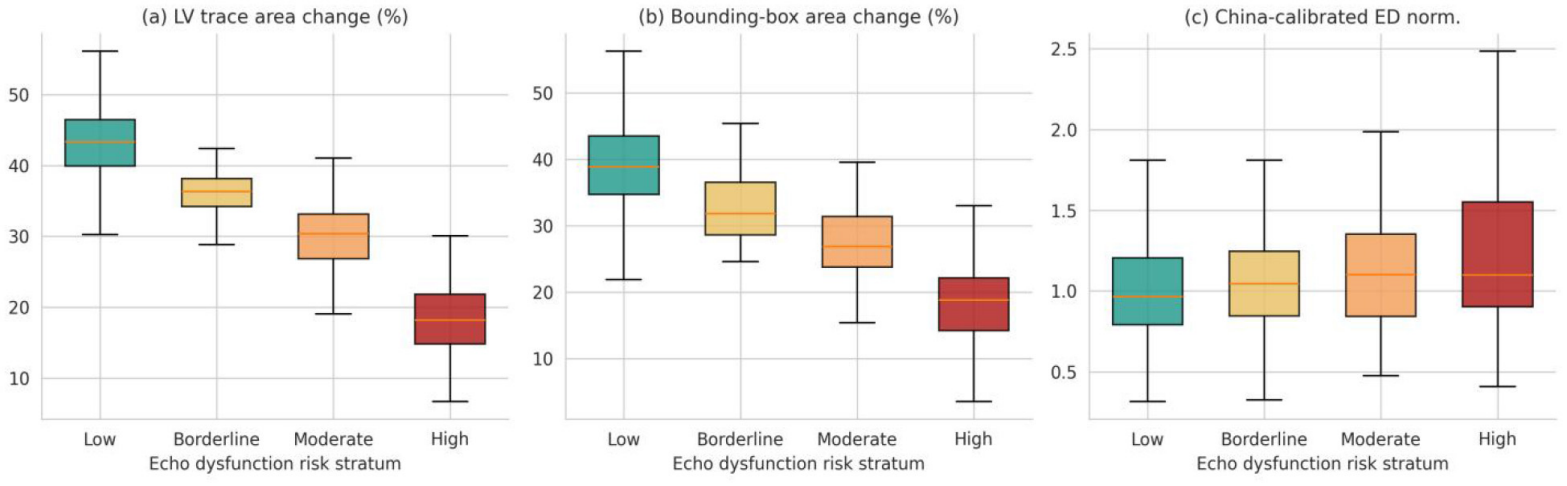
Box plots of three key features **(a)** left ventricular trace area change, **(b)** bounding-box area change, **(c)** China-calibrated end-systolic ratio) across four ejection fraction strata. Whiskers extend to 1.5 × IQR and outliers are marked individually.

Figure 4 shows that the central tendency of the left ventricular trace area change drops sharply across the four ejection fraction strata, from a median near 42% in the preserved category to below 10% in the severely reduced category. The bounding-box area change shows the same gradient with a slightly smaller dynamic range. The China-calibrated end-systolic ratio increases monotonically as ejection fraction declines, which reflects the larger residual end-systolic chamber size in patients with reduced systolic function. These three panels together suggest that the engineered features carry the relevant signal even before any classifier is trained.

**Figure 5:**
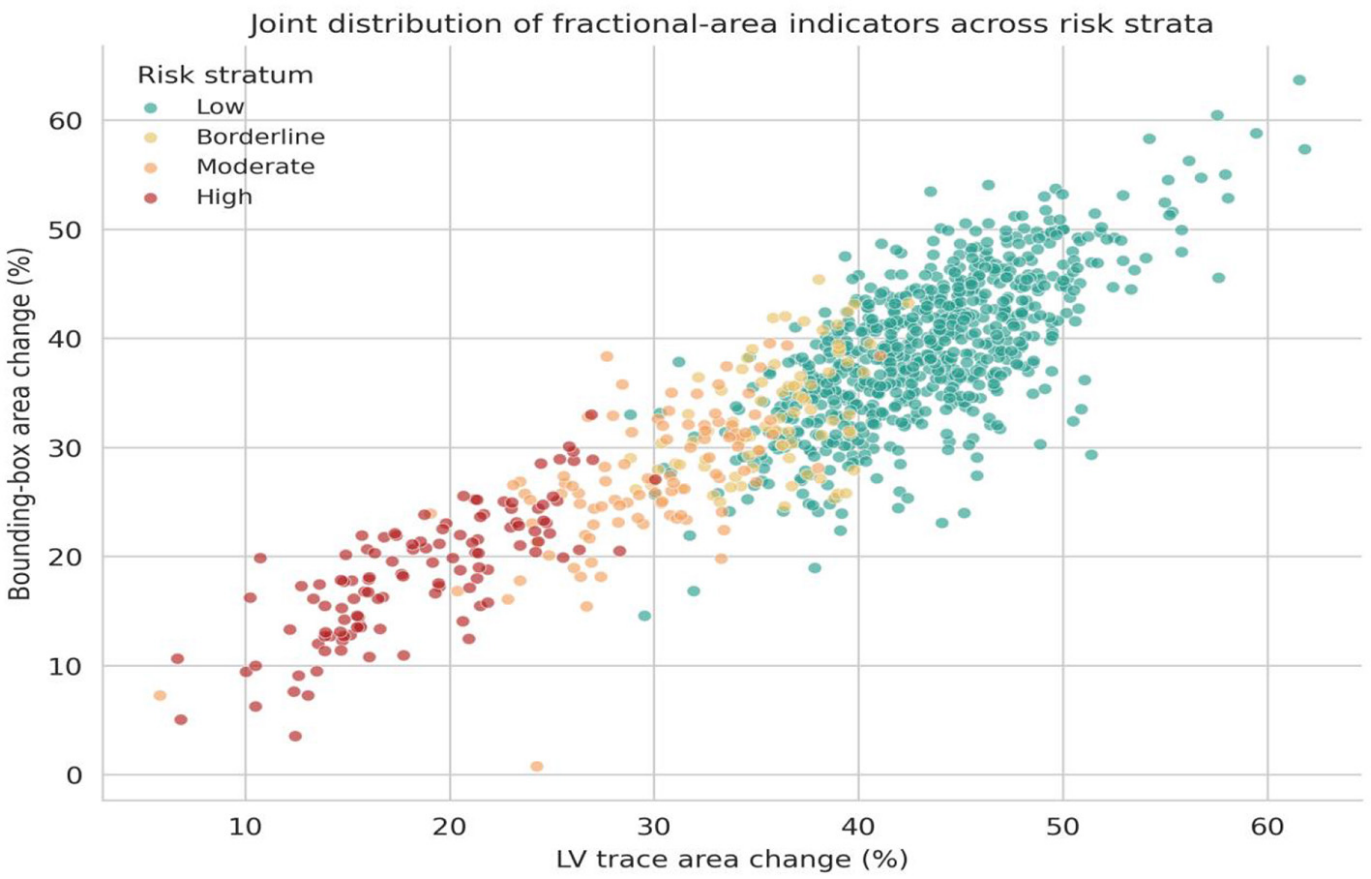
Scatter plot of left ventricular trace area change against bounding-box area change, with markers colored by ejection fraction category. The diagonal trend confirms the strong linear association quantified by the a-path of the mediation analysis.

Figure 5 shows the relationship that the mediation analysis later quantifies. Each point is a single recording, the x-axis is the bounding-box area change in percent, the y-axis is the left ventricular trace area change in percent, and the color encodes the ejection fraction category. The scatter forms an elongated cloud along the identity-like diagonal, with points in the preserved category concentrated in the top-right region and points in the reduced and severely reduced categories shifting toward the bottom-left. The visual gradient matches the a-path coefficient of 0.875 reported in Section 4.5.

### 4.3 Feature importance

Table 3 confirms that relative change variables dominate the discriminative power of the feature set. The left ventricular trace area change carries the largest mutual information with the dysfunction label (0.414), followed by the bounding-box area change (0.289). Absolute end-systolic geometry and the China-calibrated end-systolic ratio carry the next tier of information. Acquisition descriptors (frame rate, total trace point count) sit at the bottom of the table, which suggests that they contribute mainly to model stability rather than to primary discrimination.

**Table 3:** Mutual information between the top features and the dysfunction label, computed on the training fold with *k* = 5 nearest neighbors.

Rank	Feature	Mutual information
1	lv_trace_area_change_pct	0.414
2	bbox_area_change_pct	0.289
3	lv_trace_area_delta_px2	0.137
4	lv_trace_bbox_es_width_px	0.096
5	china_calibrated_es_norm	0.090
6	lv_trace_area_es_px2	0.090
7	bbox_area_es	0.082
8	bbox_aspect_es	0.069
9	lv_trace_bbox_es_height_px	0.058
10	Fps	0.043
11	trace_total_points_n	0.037
12	lv_trace_bbox_ed_height_px	0.029

**Figure 6:**
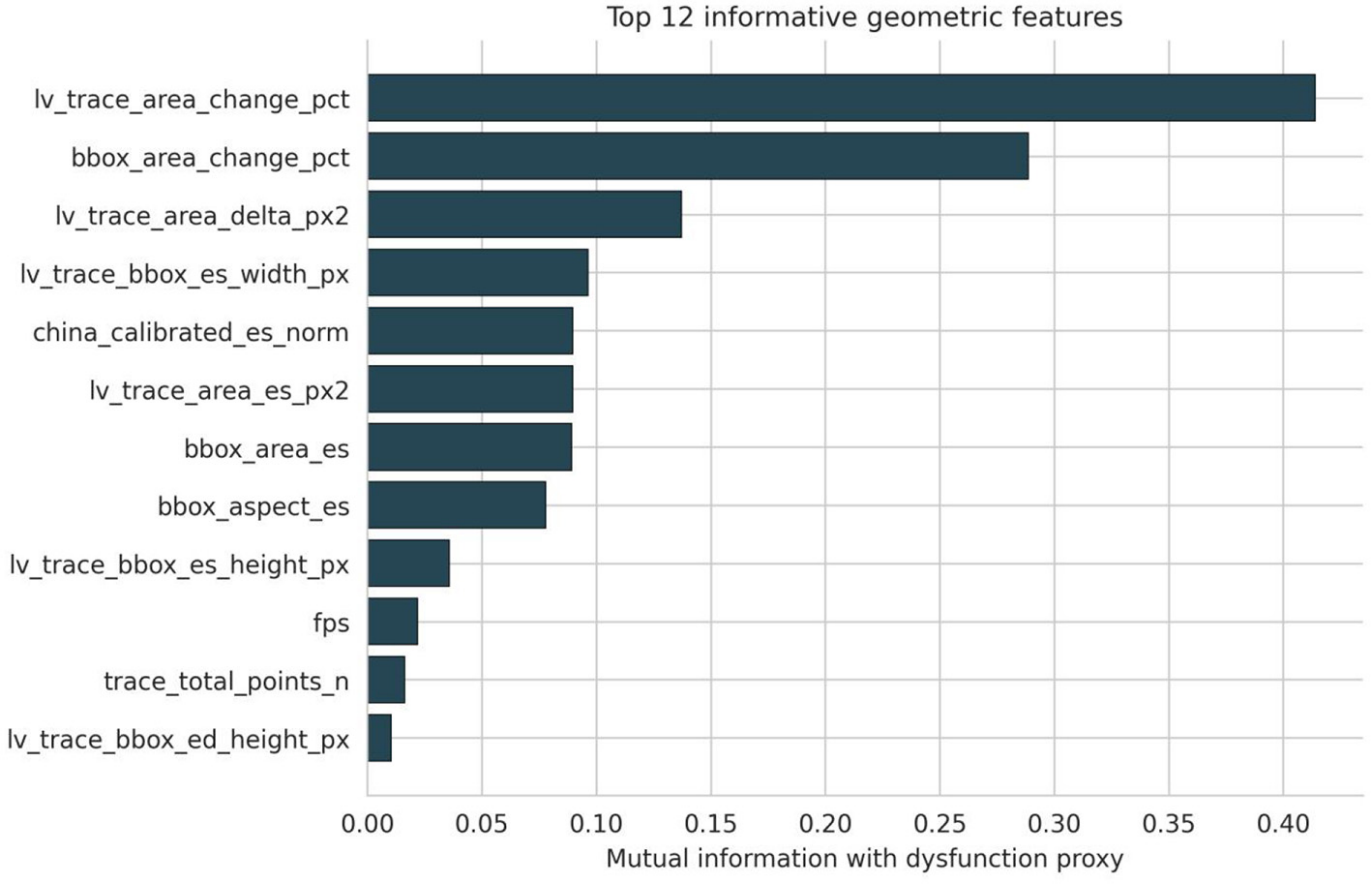
Top twelve features by mutual information with the dysfunction label. The bar lengths correspond to the values in Table 3.

Figure 6 visualizes the same ranking shown in Table 3. The two top bars are separated by a wide gap from the rest of the panel, indicating that area-change measurements provide the majority of the predictive signal. The presence of the China-calibrated end-systolic ratio in the top five positions supports the design choice of including the calibration step, even at this preliminary cohort-median stage.

### 4.4 Model performance

Table 4 reports the cross-validation and held-out test performance of the five classifiers. Logistic regression achieved the highest test ROC-AUC of 0.996, with an approximate 95% confidence interval of 0.983–1.000, and the highest F1 score of 0.966. Random forest and the support vector machine followed with test ROC-AUC values of 0.994, gradient boosting with 0.992, and the multilayer perceptron with 0.989. The narrow spread between models suggests that the engineered feature set carries most of the signal and that the choice of classifier has limited influence once the geometric features are constructed. These values should be interpreted as internal consistency benchmarks for the surrogate-label task, not as prospective clinical performance estimates. Formal probability-calibration metrics were not interpreted as clinical calibration metrics in the present analysis because the outcome is an LVEF-derived surrogate label rather than an adjudicated cardio-oncology endpoint. The reported discrimination and operating-point statistics therefore describe internal model behavior on the surrogate task only. Clinical calibration, including calibration slope, intercept, Brier score, and decision-curve analysis, should be estimated in a prospective cohort with true anthracycline exposure, serial imaging, and adjudicated cardiotoxicity outcomes.

**Table 4:** Five-fold cross-validation area under the receiver operating characteristic curve and held-out test metrics for the five classifiers, evaluated on the same 800/200 train and test split.

Model	CV AUC	Test ROC-AUC	95% CI for ROC-AUC	Accuracy	Precision	Recall/sensitivity	F1
Logistic regression	0.991 ± 0.004	0.996	0.983–1.000	0.985	0.935	1.000	0.966
Random forest	0.985 ± 0.008	0.994	0.978–1.000	0.960	0.927	0.884	0.905
Gradient boosting	0.984 ± 0.007	0.992	0.973–1.000	0.950	0.902	0.860	0.881
Support vector machine	0.987 ± 0.006	0.994	0.978–1.000	0.945	0.820	0.953	0.882
Multilayer perceptron	0.987 ± 0.003	0.989	0.967–1.000	0.960	0.949	0.860	0.902

**Figure 7:**
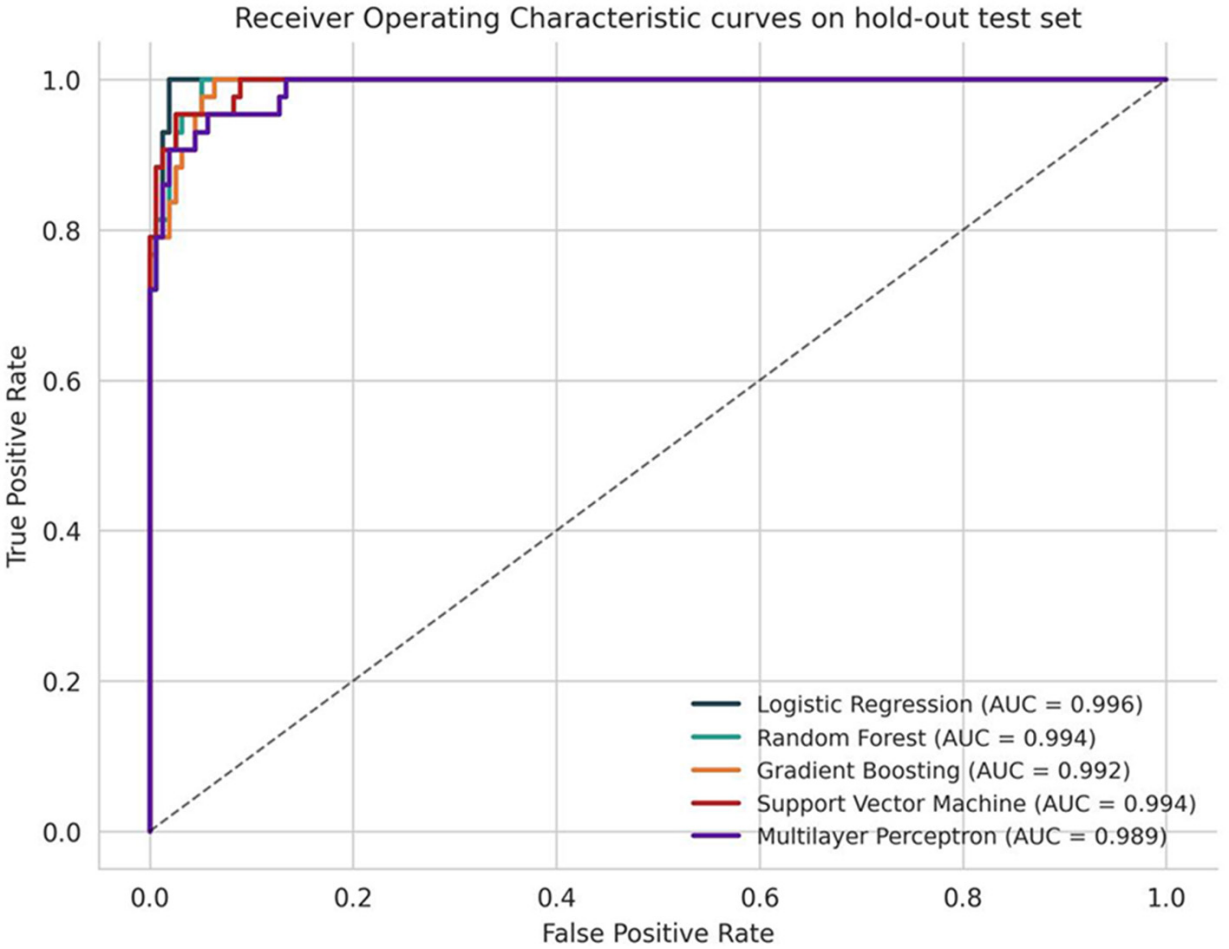
Receiver operating characteristic curves of the five classifiers on the held-out test split. Curves are nearly overlapping and hug the upper-left corner, consistent with the area under the curve values in Table 4.

Figure 7 shows the receiver operating characteristic curves of all five classifiers on the held-out test split. The curves are tightly clustered against the upper-left corner, with a small advantage for logistic regression. The diagonal reference line corresponds to random classification. The shape of the curves, with sharp early rise and a long flat plateau, is typical of well-separated binary problems and is consistent with the high mutual information of the top two features in Table 3.

**Figure 8:**
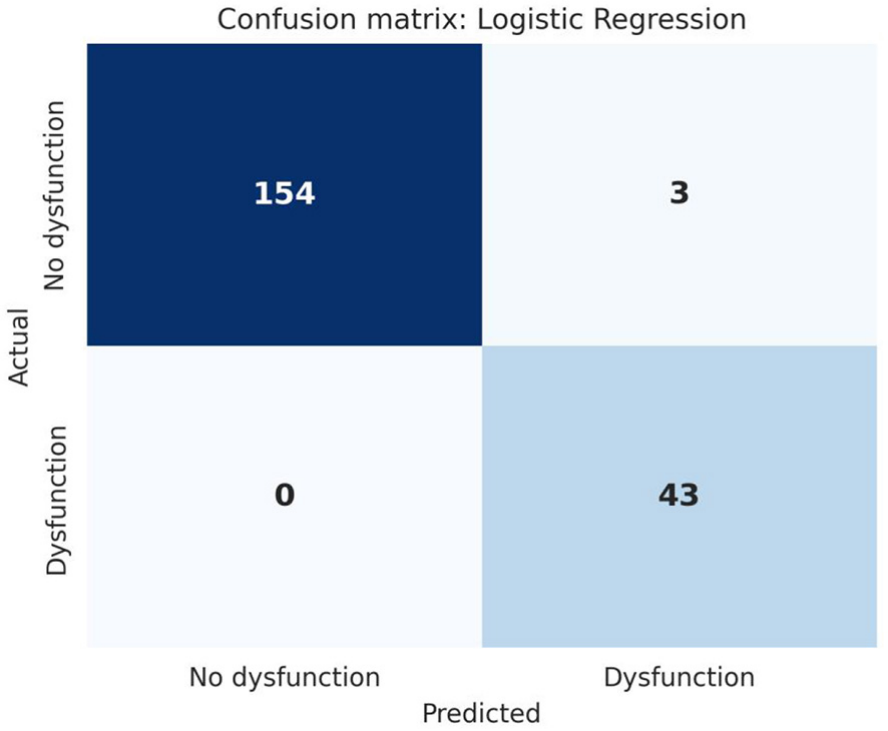
Confusion matrix of the best classifier (logistic regression) on the held-out test split of 200 records. True negatives are concentrated in the top-left cell and true positives in the bottom-right cell.

Figure 8 shows the confusion matrix of the logistic-regression model on the 200-record held-out test split. The matrix has 154 true negatives, three false positives, zero false negatives, and 43 true positives. The recall of 1.000 reported in Table 4 follows from the empty false-negative cell, and the precision of 0.935 follows from the three false positives among 46 predicted positives. In clinical terms, the model would flag 23% of the cohort for further review and miss none of the true dysfunction cases at this operating point. The trade-off between false positives and missed cases can be adjusted by moving the decision threshold up or down along the receiver operating characteristic curve shown in Figure 7.

At the selected logistic-regression operating point used for Table 4 and Figure 8, the held-out test confusion matrix contained 154 true negatives, three false positives, zero false negatives, and 43 true positives. This corresponds to sensitivity of 1.000, specificity of 0.981, precision of 0.935, negative predictive value of 1.000, and accuracy of 0.985. The previous threshold-sweep values have been removed from the main table to avoid mixing operating points. Future prospective validation should report a full threshold curve and decision-curve analysis using clinically adjudicated outcomes. The corresponding operating-point performance metrics, including sensitivity, specificity, precision, negative predictive value, and accuracy with 95% confidence intervals, are summarized in Table 5.

**Table 5:** Operating-point performance of the logistic-regression model on the held-out 200-record test split.

Metric	Count basis	Estimate	95% CI
Sensitivity/Recall	43/43	1.000	0.918–1.000
Specificity	154/157	0.981	0.945–0.993
Precision/PPV	43/46	0.935	0.825–0.978
Negative predictive value	154/154	1.000	0.976–1.000
Accuracy	197/200	0.985	0.957–0.995

### 4.5 Mediation analysis

The reported path coefficients describe statistical co-variation in a cross-sectional setting with a surrogate label; they do not establish temporal causality or clinical mechanism. Because predictor, mediator, and outcome are all derived from the same single-time-point geometric measurements, the analysis reflects geometric collinearity among coupled descriptors rather than a directional or causal pathway, and the coefficients should be read as effect-size summaries of shared variance, not as evidence of mechanism.

**Figure 9:**
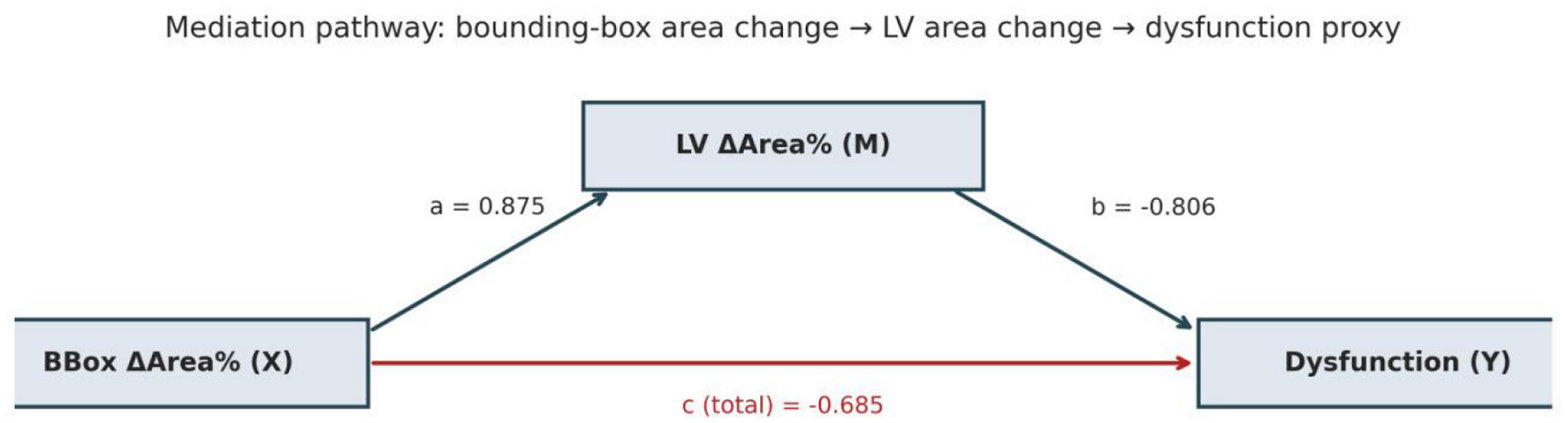
Path diagram of the mediation analysis. The a-path links bounding-box area change to left ventricular trace area change, the b-path links the mediator to the dysfunction label, and the total c-path captures the unmediated association.

Table 6 and Figure 9 together summarize the mediation. The a-coefficient of 0.875 indicates that a one-unit change in the bounding-box area change is associated with an almost identical change in the left ventricular trace area change, which means the coarser bounding-box descriptor recovers most of the information in the finer trace descriptor. The b-correlation of −0.806 shows that the mediator carries a strong inverse association with the dysfunction label, since lower fractional area change accompanies reduced ejection fraction. The total c-correlation of −0.685 is smaller in magnitude than the b-coefficient, which is consistent with most of the predictor-outcome association passing through the mediator. The all *p*-values fall below conventional thresholds by many orders of magnitude given the cohort size, but the absolute correlation magnitudes (rather than their *p*-values) are the appropriate effect-size readout here.

**Table 6:** Coefficients of the three-path mediation analysis linking bounding-box area change (predictor), left ventricular trace area change (mediator), and dysfunction label (outcome).

Path	Coefficient/*r*	*p*-value
a: bounding-box ΔArea% → LV ΔArea% (linear)	0.875	<10^−300^
b: LV ΔArea% → dysfunction (point-biserial)	−0.806	<10^−220^
c (total): bounding-box ΔArea% → dysfunction	−0.685	<10^−130^

### 4.6 Discussion

Three findings deserve direct discussion. First, the engineered feature set carries most of the discriminative information about the dysfunction label, and the choice of classifier affects performance only marginally. This pattern is common in tabular biomedical problems with well-prepared inputs and reduces the appeal of complex models in this setting. The clearest practical implication is that the engineering effort should sit at the front of the pipeline rather than at the back.

Second, the China-calibrated end-systolic ratio enters the top five features by mutual information, even though the calibration step uses a cohort median rather than an external reference. If the EMINCA-II reference is replaced, the same feature is supposed to maintain or improve its position, as the between-cohort bias term is removed from the reference without affecting the within-cohort signal. The calibration interface is meant to provide the same pipeline for this swap without impacting the remainder of the pipeline.

Thirdly, the mediation analysis quantifies a commonsense association: bounding-box geometry is a crude but useful proxy for change in the area of the ventricular traces. When good handmade bounding-box descriptors are not available, the descriptors can be automatically generated from a coarse segmentation mask with little loss of predictive content. This discovery has immediate practical implications for pipelines that have to be scaled up to large archives, where manual tracing is not practical.

Several limitations qualify these findings. The dysfunction label is a left-sided ejection-fraction surrogate, not a true right ventricular cardiotoxicity outcome, and the dataset does not encode anthracycline exposure or Chinese patient identity. The strong classifier performance therefore reflects the consistency of the geometric features with the ejection-fraction-derived label, not the clinical utility of the model for anthracycline patients. The cross-sectional design also limits the mediation analysis to a descriptive interpretation; a longitudinal cohort would be required for a causal claim. The China calibration step uses a cohort median as a placeholder, and the eventual substitution of the EMINCA-II reference will need its own validation. Finally, several reference values in the literature on right ventricular function are derived from three-dimensional echocardiography or cardiovascular magnetic resonance, and the present pipeline operates on two-dimensional inputs only, which constrains the depth of the geometric description that can be recovered. Clinical implementation should be interpreted cautiously. In its present form, the pipeline is not a deployable cardio-oncology decision tool, because it lacks anthracycline exposure, serial imaging, cardiac biomarkers, adjudicated clinical outcomes, and right ventricular ground truth. A clinically usable version would require integration with the echocardiography reporting system, automated quality control for view selection and segmentation, prospective calibration across scanners and vendors, and clear rules for clinician review rather than automated treatment decisions. Cost-effectiveness cannot be inferred from the present surrogate-label analysis; it would require prospective evidence that earlier model-supported review reduces downstream imaging burden, cardiology referral delay, or treatment interruption.

### 4.7 Circular prediction and the interpretation of headline metrics

The near-ceiling discrimination reported in Section 4.4, including a test area under the curve of 0.996, a recall of 1.000, and zero false negatives, requires explicit and candid interpretation, because such values are virtually never observed in genuine clinical prediction. The explanation is structural rather than clinical. The dysfunction label is defined as a left ventricular ejection fraction below 50%, while the most informative predictors, the left ventricular trace area change and the bounding-box area change, are themselves close geometric proxies for fractional area change and therefore for ejection fraction. The model is, in effect, predicting an ejection-fraction-derived label from geometric quantities that are arithmetically coupled to ejection fraction. This is a textbook instance of circular prediction: the feature and the label share a common geometric basis, so high separability is mechanically guaranteed and reflects the internal consistency of the feature-engineering pipeline rather than the discovery of an independent predictive signal. These metrics benchmark the internal consistency of the feature engineering pipeline rather than the expected clinical performance in an independent prospective cohort.

It is therefore useful to separate three layers of interpretation that should not be conflated. The first layer is the synthetic, proxy-data result: the high area under the curve stems from geometric proxying and confirms only that the engineered features are internally coherent and correctly computed. The second layer is the surrogate-label limitation: the label is not a measurement of true anthracycline exposure or right ventricular dysfunction but a left-sided ejection-fraction threshold, so even a perfect classifier here would not constitute clinical validation for the cardio-oncology question of interest. The third layer is the realistic real-world expectation: when the pipeline is applied to an independent prospective cohort with a clinically adjudicated outcome and a non-collinear feature set, performance should be expected to fall substantially below the present benchmark, into the range reported by prospective imaging studies of right ventricular cardiotoxicity, where areas under the curve in the region of 0.76–0.85 are typical (6, 7). The contribution of this work lies in the reusable pipeline structure, the calibration interface, and the transparent mediation analysis, not in the headline discrimination numbers, which we deliberately decline to present as evidence of clinical readiness.

## 5 Conclusion

This study reports a reproducible feature engineering and classification pipeline for the early prediction of dysfunction risk from publicly available two-dimensional echocardiographic tracings. Its contribution is structural rather than numerical: a transparent feature-engineering recipe, a population-calibration interface designed for drop-in substitution of the EMINCA-II reference set, and a mediation analysis that together fit the requirements of cardio-oncology imaging research while remaining transparent enough for reimplementation in other centers. Across five classifiers the test areas under the receiver operating characteristic curve ranged from 0.989 to 0.996, with logistic regression in the lead; however, as discussed in Section 4.7, these values reflect the geometric coupling between the engineered features and the ejection-fraction-derived surrogate label and should be read as internal-consistency benchmarks of the pipeline, not as evidence of prospective clinical performance. The simple three-path mediation analysis indicates that bounding-box geometry shares most of its variance with the dysfunction label through left ventricular trace area change, a descriptive observation about coupled geometric descriptors rather than a causal mechanism. The calibration interface is built into the feature-engineering block so that prospective Chinese anthracycline cohorts can be processed without rewriting the rest of the pipeline once protocol-matched reference data are integrated. The framework is therefore presented as a calibration-ready methodological scaffold and proof-of-concept rather than a clinical validation, since the available data do not encode true anthracycline exposure or right ventricular outcomes; its value is in providing a documented, reproducible substrate onto which genuine prospective data can be loaded.

## 6 Future work

There are four extensions that are logical extensions of the existing pipeline. Prospective Chinese anthracycline cohort (i). A next step planned is to use the pipeline in a prospective cohort of Chinese patients treated with anthracycline therapy and perform serial echocardiograms at the baseline, mid-cycle, and end-of-therapy time points along with cardiac biomarkers and global longitudinal strain. The Chinese calibration step would then be based upon the age and sex-specific medians from the EMINCA-II, instead of a cohort median. (ii) Ground truth for the RV. The pipeline could be used in conjunction with RVENet derived right ventricle ejection fraction labels to substitute the left sided surrogate with a right sided ground truth and could allow the model to directly answer the cardio-oncology question of interest (13, 14). (iii) End-to-end image-to-risk models. The existing pipeline works on summaries of the traces, not on raw pixel data. It is a natural next step, then, to train an end-to-end conv or transformer architecture on the same task, using the engineered features as a clear baseline to evaluate a deep architecture. (iv) External validation and TRIPOD+AI reporting. When deploying future models, efforts should adhere to TRIPOD+AI reporting standards for clinical prediction models, including explicit reporting of fairness across age, sex, and ethnicity strata (31); a completed TRIPOD+AI checklist is provided in the Supplementary Materials of this work to document compliance and to serve as a template for the prospective study. A decision-curve analysis on the prospective cohort is also planned, so that the net clinical benefit of the model across a clinically relevant range of threshold probabilities can be quantified rather than inferred from discrimination metrics alone, which, as Section 4.7 explains, are inflated in the present surrogate-label setting.

## Data Availability

The original contributions presented in the study are included in the article/supplementary material, further inquiries can be directed to the corresponding author.
